# Ethical, Legal, and Social Implications of Gene‐Environment Interaction Research

**DOI:** 10.1002/gepi.22591

**Published:** 2024-09-24

**Authors:** Stephanie Calluori, Kaitlin Kirkpatrick Heimke, Charlisse Caga‐anan, David Kaufman, Leah E. Mechanic, Kimberly A. McAllister

**Affiliations:** ^1^ Columbia Mailman School of Public Health New York New York USA; ^2^ Division of Genome Sciences NHGRI Bethesda Maryland USA; ^3^ Genomic Epidemiology Branch, Epidemiology and Genomics Research Program, DCCPS, NCI Bethesda Maryland USA; ^4^ Division of Genomics and Society NHGRI Bethesda Maryland USA; ^5^ Genes, Environment, and Health Branch, Division of Extramural Research and Training NIEHS Durham North Carolina USA

**Keywords:** environmental justice, ethical, gene‐environment, legal, social

## Abstract

Many complex disorders are impacted by the interplay of genetic and environmental factors. In gene‐environment interactions (GxE), an individual's genetic and epigenetic makeup impacts the response to environmental exposures. Understanding GxE can impact health at the individual, community, and population levels. The rapid expansion of GxE research in biomedical studies for complex diseases raises many unique ethical, legal, and social implications (ELSIs) that have not been extensively explored and addressed. This review article builds on discussions originating from a workshop held by the National Institute of Environmental Health Sciences (NIEHS) and the National Human Genome Research Institute (NHGRI) in January 2022, entitled: “Ethical, Legal, and Social Implications of Gene‐Environment Interaction Research.” We expand upon multiple key themes to inform broad recommendations and general guidance for addressing some of the most unique and challenging ELSI in GxE research. Key takeaways include strategies and approaches for establishing sustainable community partnerships, incorporating social determinants of health and environmental justice considerations into GxE research, effectively communicating and translating GxE findings, and addressing privacy and discrimination concerns in all GxE research going forward. Additional guidelines, resources, approaches, training, and capacity building are required to further support innovative GxE research and multidisciplinary GxE research teams.

## 1 Introduction

Many health conditions are affected by a complex combination of genetic and environmental risk factors. Gene‐environment (GxE) interaction research explores the varying effect of environmental exposure(s) given an individual's genetic and epigenetic background for risk of complex disorders (Ritz et al. 2017). (See Table 1 for detailed definitions/explanations of ethical, legal, and social implications [ELSI] relevant terms used throughout this text). Genetic variation in human populations is known to impact the entry, absorption, activation, and detoxification of many environmental chemicals, which ultimately impacts human health outcomes (Christiani et al. 2001). A growing number of replicated GxE interactions are now linked to a wide range of human diseases and health conditions (Virolainen et al. 2023). Examples include *NAT2* and smoking for bladder cancer, *PON1* and pesticide exposure for Parkinson's disease, *NOS2* and traffic pollution for respiratory disorders, and *BRCA‐1* associated protein‐1 (*BAP1*) mutations and asbestos exposure for mesothelioma (Ritz et al. 2017; Motsinger‐Reif et al. 2024).

**Table 1:** Glossary of gene‐environment interactions (GxE) ethical, legal, and social implications (ELSI) terms used throughout the article.

Clinical Laboratory Improvement Amendments of 1988 (CLIA)	Amendment to the Public Health Services Act, which revised the federal program for certification and oversight of clinical laboratory testing. CLIA regulations establish quality standards for laboratory testing performed on specimens from humans for the purpose of diagnosis, prevention, or treatment of disease, or assessment of health. CLIA regulations generally apply to patient‐specific results and usage in clinical care.	https://www.cdc.gov/clia/law-regulations.html Laurino et al. (2017). Bookman et al. (2006).
Community‐ engaged research	Continuum of approaches to involve the community in the research process, ranging from community consultation to community driven research. Community‐based participatory research (CBPR) is one very important example, involving authentic, collaborative partnerships between researchers and community‐based organizations, based on principles of equity, co‐learning, mutual benefit, and long‐term commitment.This approach incorporates community theories, participation, and practices into the research efforts so that community members are involved in all aspects of the research and help drive the research questions being asked.	Haboush‐Deloye et al. (2023). Key et al. (2019). Wallerstein and Duran (2006).
De‐identification	The process of reducing data content to decrease the likelihood of discovering a person's identity.	El Emam, Rodgers, and Malin (2015).
Environmental health literacy	The knowledge and skills that prepare people to make health‐protective decisions using available environmental data.	Finn and O'Fallon (2017). Gray and Martin (2019)
Environmental justice	Just treatment and meaningful involvement of all people, regardless of income, race, color, national origin, Tribal affiliation, disability, sexual orientation, sex, or gender identity, with respect to the development, implementation, and enforcement of environmental laws, regulations, and policies.	EPA: https://www.epa.gov/environmentaljustice DCPD‐202300319—Executive Order 14096‐Revitalizing Our Nation's Commitment to Environmental Justice for All: https://www.govinfo.gov/app/details/DCPD-202300319
Gene‐environment interaction (GxE)	Broadly refers to the varying effect of environmental exposure(s) on risk of complex disorders given an individual's genetic/epigenetic background, or conversely, the varying impact of a genetic/epigenetic variant on risk of complex disorders given an individual's environmental exposure(s).	NHGRI: https://www.genome.gov/genetics-glossary/Gene-Environment-Interaction NIEHS: https://www.niehs.nih.gov/news/events/pastmtg/2022/elsi/index.cfm
Genetic Information Nondiscrimination Act (GINA)	Federal legislation in the United States that protects individuals against discrimination based on their personal genetic information, as it applies to health insurance and employment.	NHGRI: https://www.genome.gov/genetics-glossary/Genetic-Information-Nondiscrimination-Act
Geospatial measures (geomarkers)	Measures used to specify a particular geographic position on Earth and often used to estimate past and current exposures linked to different health outcomes.	Rasnick et al. (2023).
Health disparities	Health differences that are closely linked with social, economic, and/or environmental disadvantage (i.e., disproportionately exposed to environmental hazards). Health disparities adversely affect groups of people who have systematically experienced greater obstacles to health based on their racial or ethnic group; religion; socioeconomic status; gender; age; mental health; cognitive, sensory, or physical disability; sexual orientation or gender identity; geographic location; or other characteristics historically linked to discrimination or exclusion.	Healthy People 2030: https://health.gov/healthypeople/priority-areas/health-equity-healthy-people-2030
Health Insurance Portability and Accountability Act (HIPAA)	A federal law that required the creation of national standards to protect sensitive patient health information from being disclosed without the patient's consent or knowledge.	CDC: https://www.cdc.gov/phlp/publications/topic/hipaa.html
HIPAA Safe Harbor	One of two methods to achieve de‐identification in accordance with the HIPAA Privacy Rule. Involves removal of 18 types of identifiers (e.g., names, social security number, medical record numbers) and requires that the covered entity does not have knowledge that the remaining information could be used, alone or in combination with other information, to identify the individual.	https://www.hhs.gov/hipaa/for-professionals/privacy/special-topics/de-identification/index.html
Protected Health Information (PHI)	Individually identifiable health information, including demographic information, which relates to past/present or future physical or mental health condition, provision of healthcare, or payment for the provision of healthcare.	HIPAA: https://www.hhs.gov/hipaa/for-professionals/privacy/special-topics/de-identification/index.html#standard
Report back	Return of research results to an individual research participant and/or a larger community. Both types of reporting back will often provide a comparison to help research participants contextualize their personal exposure or the community‐level risk.	Lebow‐Skelley et al. (2020).
Social determinants of health (SDOH)	The conditions in which people are born, grow, learn, work, play, live, and age, as well as the wider set of structural factors shaping the conditions of daily life. Structural factors include social, economic, and legal forces, policies, and systems (e.g., discriminatory systems like racism and sexism) that determine opportunities and access to such factors as high‐quality jobs, education, housing, and health care.	NIH‐wide Social Determinants of Health Research Coordinating Committee: https://www.ninr.nih.gov/researchandfunding/nih-sdohrcc#tabs2
Team science	Research conducted in an interdependent fashion by more than one individual. Teams may be of one discipline, multidisciplinary, or interdisciplinary.	chrome‐extension://efaidnbmnnnibpcajpcglclefindmkaj/https://nap.nationalacademies.org/resource/19007/dbasse_165410.pdf (https://www.nationalacademies.org/our-work/the-science-of-team-science)

GxE interactions are situated within a complex ecosystem of biological, environmental, social, and structural factors that shape overall health and contribute to health disparities (Diez Roux 2011). Understanding GxE interactions will provide critical information about exposure pathways and disease mechanisms with the potential to impact health at the individual, community, and population levels (Thomas 2010; McAllister et al. 2017). GxE research will inform disease treatment and management, preventative and screening measures, as well as public health interventions, regulations, and policies. These far‐reaching, multi‐level implications demonstrate the potential of GxE findings to impact social and environmental justice in health and to raise complex ethical, legal, and social implications (ELSI) as this field evolves.

While there is a long history of research examining ELSI within genetics research and some within environmental health research, little has been done examining unique ELSI within GxE research. One common GxE example is that individuals with certain *GSTP1* (glutathione S‐transferase P1) gene variants are known to be more likely to have asthma when exposed to certain air pollutants (Morales and Duffy 2019). How would this knowledge inform clinical and public health applications? How would this knowledge affect how one prioritizes action on air pollutants associated with this GxE finding? Should regulations be set for all on the basis of protecting the most genetically susceptible to environmental exposures in a population (Christiani et al. 2001)? How does one explain a GxE scenario to a lay audience or communicate this concept to affected populations? How should these findings be reported back to participants in a research study? Many challenges associated with genomic and environmental literacy and report back of results are emerging with GxE findings such as these, beyond the already complex issues associated with genetic or environmental exposure results alone. Is it beneficial for an individual to know of their susceptibility to a chemical in combination with a genetic variant if the source of the chemical exposure is unknown (and therefore unactionable)? Additional ethical concerns related to environmental justice, environmental health disparities, and potential stigmas or discrimination could arise with respect to GxE findings. Lastly, many GxE findings manifest as an epigenetic/epigenomic change. The report back of epigenomic findings presents additional ELSI issues due to the potential reversibility of these changes and the possible transgenerational effects of some epigenomic modifications (Ribatti 2021).

To further explore ELSI considerations for GxE research, the National Institute of Environmental Health Sciences (NIEHS) and the National Human Genome Research Institute (NHGRI), with participation from the National Cancer Institute (NCI), hosted the National Institutes of Health (NIH) workshop entitled: “Ethical, Legal, and Social Implications of Gene‐Environment Interaction Research” on January 11–12, 2022 (“NIEHS” 2023a). Several major themes were discussed including: (1) Opportunities/approaches to augment collaborations with communities and address community concerns; (2) Unique social and environmental justice issues associated with identification of higher‐risk populations in GxE studies; (3) Complexities of reporting back individual and community level findings and risks from GxE research; and (4) Privacy, discrimination, and legal concerns associated with identification of subpopulations at higher disease risk due to genetic susceptibilities of environmental exposures. Here, we highlight and expand upon these themes and provide broad recommendations for addressing unique ELSI in GxE research. The authors are indebted to the workshop's speakers, panelists, and moderators for guiding thinking around the concepts in this review manuscript.

## 2 Establish Sustainable Community Partnerships

### 2.1 Role of Community in GxE Research

Establishing authentic and sustainable community‐empowering partnerships, where communities are equitable partners in the research enterprise, is key to conducting innovative and impactful GxE research. Communities are uniquely equipped to define research priorities and inform the research process as they have the lived experience of factors impacting their health and often have intimate knowledge of the resources needed to address environmental injustices (Van Horne et al. 2023). As such, community members should be integral members of the research team, meaningfully engaged in each step from research planning to implementation. Researchers should take steps to build trust and cultivate transparency. Expectations for the ways in which community members, individually and collectively, will benefit from participating in the study should be discussed early and clearly communicated to everyone on the research team. Communities that have been underserved are impacted by health disparities that both result from and perpetuate social and environmental injustices (Senier et al. 2017) (See Table 1 for detailed definitions/references surrounding health disparities). Researchers should be responsive to and collaborate with communities in ways that center the knowledge and voices of the community and are sensitive to these environmental injustices. This starts with researchers and communities forming meaningful relationships before the study to gain a deep understanding of communities' experiences, priorities, and concerns, as well as the researchers' scientific areas and proposed hypotheses of interest.

### 2.2 Lessons and Best Practices From Genetic/Genomics and Environmental Health Research

Previous efforts by scientists to engage with communities in both genomics and environmental health research provides important lessons and best practices for GxE research. For example, the Breast Cancer and the Environment Research Centers (BCERC) incorporates outreach and translation cores, to move beyond recruitment and retention and to support bidirectional feedback with communities. This project involves a diverse nation‐wide cohort predominantly comprised of African American and/or Hispanic participants (Hiatt et al. 2009). This research team, which included community partners, recognized the need to work closely with a broad range of relevant stakeholders, such as families, advocates, and public health officials, to address unexpected challenges (Hernick et al. 2011). Similar principles were applied in “Project Creating a Higher Understanding of Cancer Research and Community Health” (CHURCH), which established collaborative partnerships with faith‐based organizations in Houston to engage African American communities as partners in cancer prevention research (McNeill et al. 2018). In addition, successful genomics research partnerships with Indigenous communities have involved the following: open, ongoing discussion with the community; participation of tribal agencies; recognition of tribal sovereignty and local knowledge by researchers; inclusion of community members as part of the research team; a community‐formed and negotiated data sharing process; educational opportunities and mutual learning; community approval before publishing results; and increased research capacity in Indigenous communities (e.g., supporting Indigenous scientists) (Claw et al. 2018; Garrison et al. 2019; Blacksher et al. 2021). Furthermore, both genetic and environmental health researchers have recognized that diverse perspectives are critical in producing better science (“Science Benefits From Diversity” 2018; Freeman and Huang 2014). Research teams that reflect the diversity of study participants also strengthen trust and engagement in research. In environmental health research, community partnerships have also advanced understanding of environmental health contributors in communities and challenged researchers to engage in a more comprehensive understanding of disease causation and probable solutions.

### 2.3 Needs for Community‐Driven GxE Research

Conducting community‐engaged research requires sustainable infrastructures to form and support long‐term collaborations (See Table 1 for detailed community‐engaged research approaches and descriptors). Support is also essential for capacity building in terms of strengthening research infrastructure in communities and resources (e.g., education and policy actions) needed to potentially act on GxE research findings. In addition, researchers need specific training in community engagement methods (Baldwin et al. 2021). As researchers engage with communities, GxE research needs a forum for sharing best practices for community‐engaged research across studies. New funding and educational approaches in GxE research could help facilitate these efforts (Van Horne et al. 2023; Grayson, Doerr, and Yu 2020). Current programs that could inform future community partnerships in GxE research include the Community Partnerships to Advance Science for Society (ComPASS), which is focused on developing, sharing, and evaluating community‐led interventions to reduce health disparities (“Community Partnerships to Advance Science for Society (ComPASS)” 2022), and the Partnerships for Environmental Public Health (PEPH). PEPH is a network of scientists, community members and educators, health care providers, and public health and policy makers that have worked together to advance the impact of environmental health research at the individual, community, and national level (“NIEHS” 2022).

## 3 Incorporate Social Determinants of Health (SDOH) to Address Environmental Justice

### 3.1 Integration of SDOH Into GxE Research

Incorporating SDOH is a key step toward conducting impactful, rigorous, and equitable GxE research. SDOH, especially structural racism, the uneven distribution of health care resources, and the disproportionate exposure to environmental hazards, are of high interest to many communities as these factors contribute substantially to health disparities (Goldenberg et al. 2013; Link and Phelan 1995). Specifically, studying GxE interactions within the context of SDOH will generate a more comprehensive understanding of the causes of health and disease. Social and structural factors drive variation in environmental exposures (Senier et al. 2017). Understanding the larger social and structural context of environmental exposures facilitates identification of root causes and modifiable risk factors. Modifiable risk factors can be candidates for interventions, presenting pathways to practical solutions desired by communities to protect health and advance environmental justice. The examination, communication, and dissemination of GxE research findings in ways that contextualize the data within SDOH and discuss the limitations and implications of the research are important to actively counter harmful data misinterpretations that may perpetuate discrimination and inequities (Varma et al. 2023). Approaches for how to best integrate SDOH into GxE research and address potential challenges are under active development.

Integrating SDOH into GxE research will require broadening perspectives from the individual to the population level. Current studies often focus on genetics, the individual, and disease mechanisms (Ackerman et al. 2017; Darling et al. 2016). Emphasis on the individual may pose an unbalanced focus on individual responsibility for improving health (Darling et al. 2016; Ferryman and Pitcan 2018). Consideration of environmental, social, and structural exposures calls for an expansion of who bears responsibility for mitigating adverse exposures. Furthermore, an unbalanced emphasis on personalized care may narrow the benefits of prevention and treatment efforts, especially given disparities in health care access (Senier et al. 2017). Both clinical and public health applications should be emphasized to ensure that prevention, treatment, remediation, and intervention efforts benefit both the individual and the community (Ackerman et al. 2017; Darling et al. 2016; Ferryman and Pitcan 2018).

### 3.2 Methods, Approaches, and Expertise Needed for Examining SDOH in GxE Research

To facilitate the incorporation of SDOH and a public health perspective into GxE research, a multidimensional framework is needed to capture the myriad of risk factors (e.g., biological, environmental, social, structural), their complex interactions, and the multiple levels (individual, community, global) at which they operate and can be acted upon to influence health disparities (Diez Roux 2011). Several frameworks have been proposed for integrating SDOH into GxE research and the adjacent fields of environmental health and exposure science (Van Horne et al. 2023; Senier et al. 2017; Juarez et al. 2014; Zota and VanNoy 2021; “National Institute on Minority Health and Health Disparities (NIMHD)” 2023; Casey et al. 2023). Further exploring the strengths and limitations of these frameworks would be useful for the field. Overall, the use of more solution‐oriented methods, including intervention approaches and community‐engaged efforts, as well as the further development of rigorous statistical research methods related to environmental health and justice studies may provide more actionable evidence to support policy and public health changes (Casey et al. 2023).

Methods to make use of these multi‐faceted frameworks and integrate SDOH into GxE research are beginning to be developed. Progress has been made on creating measures for SDOH (Emeny et al. 2022; Juarez et al. 2014). For instance, the PhenX Toolkit provides data collection protocols for measuring SDOH at the individual and structural levels, spanning areas such as built and natural environments, structural racism, educational attainment, and access to health services (Hamilton et al. 2011; Krzyzanowski et al. 2023; “National Institute on Minority Health and Health Disparities (NIMHD)” 2020; “PhenX Toolkit: Collections” 2024). The *All of Us* research program's SDOH Survey collects a variety of social and environmental factors related to neighborhood characteristics, food and housing security, and discrimination and psychosocial stress (“National Institutes of Health All of Us Research Program” 2023). Researchers are also developing analytical strategies for incorporating social and structural factors and multiple exposures (Juarez et al. 2014; Zota and VanNoy 2021).

Interdisciplinary, collaborative research teams (or “team science”) are key to accessing the expertise needed for effectively and ethically implementing multi‐dimensional research studies that capture the complexity of factors involved in GxE research (Baldwin et al. 2021; Ackerman et al. 2017). Community engagement will be critical in forming interdisciplinary teams that will examine SDOH. In addition, behavioral and sociological expertise will be important for incorporating social and structural factors into GxE investigations (Senier et al. 2017). Bioethicists are needed as key members of research teams to explore the legal and ethical implications of GxE research questions (Ray and Cooper 2024). Diversity of perspectives will generate new approaches for conducting GxE studies that adhere to social and environmental justice principles (Ackerman et al. 2017). New funding mechanisms and educational models could help support interdisciplinary collaborations, foster interdisciplinary thinking, and cultivate interdisciplinary methods.

## 4 Communicate and Translate GxE Research Findings

### 4.1 Historical Context for Reporting Genomics and Environmental Health Research Results

For over two decades, researchers have faced the ethical dilemma of what information should be communicated back to study participants—an issue often referred to as “return” (in genomics research) or “report back” (in environmental health studies) of results. In recent years, increased evidence of potential benefits, minimal harms, and participant desire to receive results have led to prominent bodies supporting the practice of report back, such as the National Academies of Sciences, Engineering, and Medicine (NASEM) report in 2018 (“National Academies of Sciences, Engineering, and Medicine” 2018). NASEM recommends that investigators testing human biospecimens routinely consider whether and how to report back individual results and include plans in their protocols. These recommendations are rooted in principles of reciprocity, respect, and transparency. These guidelines also demonstrate the responsibility of researchers to share “right‐to‐know” information and highlight researcher/community “co‐ownership” of data. Researchers, in addition to participants, benefit from report back since this practice may increase participant recruitment and retention, as well as promote greater public trust in research (“National Academies of Sciences, Engineering, and Medicine” 2018). However, many challenges remain regarding the scope of what to report back to individuals and communities and how best to communicate findings to maximize the benefits and minimize harms.

There are distinctions between the norms and processes of returning genetics results and reporting environmental findings that can inform GxE research “report back”. There are many different types of genetic variants to report. Genetic variants that contribute to complex phenotypes include rare, highly penetrant mutations; common variants (such as those that increase susceptibility to disorders or are associated with drug response); and combinations of variants that together contribute to a polygenic risk score (Ritz et al. 2017; Black 2016; Pang et al. 2023; Ambrosone et al. 2008; Sanderson, Emery, and Higgins 2005). Each has unique considerations for reporting and interpretation. Moreover, interpretation of genetic results may be challenging for individuals of non‐European ancestry due to the European centric bias of most genetic discovery studies (Martin et al. 2019). In genetics and genomics, “return of results” has developed with investment in using genomic sequencing technologies in the clinic and advances in the interpretation of genomic findings. These results are usually focused on the individual and family unit. Multiple studies have explored how to communicate genetics and genomics results in clinical and research settings. Challenges include the number, complexity, and uncertainty of results, as well as managing participant expectations. The move towards genome and exome sequencing rather than single gene or gene panel tests further exacerbates these challenges (“National Academies of Sciences, Engineering, and Medicine” 2018; Amendola et al. 2015; Suckiel et al. 2021). Studies of genetic literacy have found gaps in knowledge about genetics and genomics (Daly and Kaphingst 2023). Additionally, health literacy as well as numeracy may impact the ability to interpret genetic results (Kaphingst et al. 2021; Drelles et al. 2021). One way that clinical research studies involving genetics have attempted to address these problems is through the consenting process; genetic counselors spend significant time with participants in the counseling session to set expectations, emphasize limitations, and prepare participants for uncertain results so that they truly understand what they are consenting to in signing up for a research study with return of genetic results (Wynn et al. 2018).

In contrast, in environmental health research, “report back” generally draws from public health monitoring and a strong history of community engagement. The emphasis is largely on reporting environmental results back in aggregate to an entire community or study population, in addition to reporting back individual results. Despite concerns that receiving environmental research findings could distress participants, minimal harms have been reported thus far (“National Academies of Sciences, Engineering, and Medicine” 2018; Ohayon et al. 2017; Brody et al. 2014). In California, individuals are legally entitled to request and receive their biomonitoring results (Brody et al. 2014; “Communicating Results: Returning Results to Participants” 2023). Along with potential public health interventions, report back of environmental exposure results may enable participants to take individual actions, such as limiting use of the exposure source, changing purchasing behaviors, or deciding to join in collective action (Oksas et al. 2022). Participants may also help researchers identify sources of exposure as part of report back (Ohayon et al. 2017).

Several challenges remain for environmental report back, including understanding the findings themselves. These challenges include the complexity of measuring whether, when, and to what extent environmental exposures occurred and how to assess complex environmental mixtures and measurement error. Current limitations on performing accurate historical place of residence analyses coupled with methodological issues on temporal lag exposure effects also make environmental exposure studies challenging (McAllister et al. 2017; Bookman et al. 2011; Hutter et al. 2013; Mechanic et al. 2012; Kraft and Aschard 2015). Researchers must account for the various types of exposures (e.g., physical, biological, and psychosocial) and the source and place of exposures (e.g., route of contact and timing, metabolism/excretion, and distribution in target tissues) when assessing impact. Furthermore, for some chemical exposures, the meaning or significance of dose levels may be uncertain, unknown, or dynamic (“National Academies of Sciences, Engineering, and Medicine” 2018). The regulatory status of chemicals can also be in flux (Goho 2016). Partly due to these complexities, there is still a need for more quality control guidance for many forms of environmental data. This contrasts with the return of genetic/genomic findings, in which confirmation in a Clinical Laboratory Improvement Amendments (CLIA) certified lab has become the gold standard for clinically reliable genomic results. (CLIA regulations generally apply to laboratories where *patient‐specific* results are reported “for the diagnosis, prevention, or treatment of disease, or assessment of health”) (“Centers for Disease Control and Prevention” 2022; Wang et al. 2023; Laurino et al. 2017; Lyon and Segal 2013). Despite these challenges, many environmental health studies have been able to report back results with careful quality control procedures, even when an official clinical standard for an “unsafe” level of the chemical being studied does not exist (e.g., new emerging chemicals of concern, such as phthalates) (Korfmacher and Brody 2023). Community input has been highly valued in these cases (“National Academies of Sciences, Engineering, and Medicine” 2018; Claudio et al. 2018; Lebow‐Skelley et al. 2020).

### 4.2 Challenges of GxE Report Back

Communication and report back of GxE findings face many of the same difficulties as those for genetic and environmental findings, as well as its own unique challenges (McAllister et al. 2017; Bookman et al. 2011; Hutter et al. 2013; Mechanic et al. 2012; Kraft and Aschard 2015). GxE disease risks are generally more nuanced than a risk established from either a highly penetrant genetic variant or an environmentally driven disease risk. This is because the GxE disease risk associated with a genetic variant may only manifest with a particular timing and dose of exposure. For example, the genetic susceptibility to exposures implicated in asthma manifest differently and involve entirely different mechanistic pathways for child versus adult‐onset asthma (Morales and Duffy 2019). In another example, the detection of the interaction of *FTO* with physical activity for obesity was highly dependent on the amount of physical activity (Ritz et al. 2017). In such cases, the communicated risk would depend on both a thorough assessment of individual genetic variation and environmental exposures. Since genetic variation is at the individual level, it is particularly challenging to communicate risk for health outcomes for studies where an environmental exposure may be measured at the community level.

Additionally, there are unique challenges with communicating epigenomic findings. GxE research and report back provide a new emphasis on these considerations because an epigenetic finding is often how a GxE interaction is initially identified at the cellular or molecular level (e.g., as a methylation, histone, or chromatin change). Epigenetic markers can change over time, with exposures, and by tissue or cell types. Thus, many questions remain for when an epigenetic biomarker should be considered in the clinical setting (Ladd‐Acosta and Fallin 2016; Santaló and Berdasco 2022). The potential reversibility of epigenetic marks by reduction in environmental exposures raises environmental justice and discrimination considerations. Epigenetic alterations are sometimes associated with individual lifestyle choices (such as smoking, physical activity, diet, and so on) that are associated with many diseases. This raises concerns regarding potential individual stigmatization where the possibility of reverting the epigenetic alterations (and therefore reducing disease risk) is presumed to be dependent on the individual's behavior. In addition, low‐income and under‐resourced communities may have less opportunity to alter their environmental exposures at either an individual or community level, increasing concerns of social discrimination or stigmatization, which highlights the need for equitable solutions in mitigating environmental hazards (Santaló and Berdasco 2022). A recent paper examined the potential ethical issues related to conducting epigenetic research on three populations (Indigenous, autistic, and transgender) where concerns related to an increase in stigmatization, racialization, or other negative impacts resulting from the research have been voiced (Saulnier et al. 2022). In addition, the potential transgenerational effects of epigenomic findings in the context of GxE raises many ELSI concerns that are just beginning to be explored, such as the possible impact of present exposures on future generations' health and how this should be addressed (Santaló and Berdasco 2022; Breton et al. 2021; Motsinger‐Reif et al. 2024).

### 4.3 Strategies for Communicating GxE Results to Participants

For GxE research, community involvement in the development and communication of report back is key to ensure report backs are useful, accessible, and, when possible, actionable. Community engagement has historically played a major role in report back for environmental health research and more recently GxE research (“National Academies of Sciences, Engineering, and Medicine” 2018; Lebow‐Skelley et al. 2020). GxE findings are critical in empowering communities to advocate for their health and advance environmental justice. Understanding participants' desires for receiving their own results is vital for effective participant engagement in research. Studies consistently demonstrate a strong preference among participants for return of results, even if there is uncertainty in the health implications or methods for risk reduction. To elicit the views of GxE study participants, researchers have used Community‐Based Participatory Research approaches (see Table 1 for detailed definition), which establish equitable collaborative partnerships between researchers and community‐based organizations and allow the community to drive the research questions being asked in their neighborhoods (Oksas et al. 2022; Tomsho et al. 2022a, 2022b). Community and participant input can guide effective communication, including what study information should be shared (e.g., appropriate level of detail) and how best to deliver it (i.e., appropriate medium) (Lebow‐Skelley et al. 2020).

Researchers are also studying how participants engage with reports of various structures and formats (Brody et al. 2021; Tomsho et al. 2019). Personal reports appear to draw more engagement from participants than reports limited to community‐level results. Some hybrid models have been successfully used to report back both individual and community levels. Reporting back results to participants should be done in an accessible way, considering the genomic/environmental health literacy of participants and disseminating findings in culturally appropriate ways (Lebow‐Skelley et al. 2020; Ramirez‐Andreotta et al. 2016). To evaluate accessibility of report back materials, researchers have looked to existing tools, such as the CDC Clear Communication Index, and have produced new tools to facilitate report back creation (Tomsho et al. 2022b; Polka et al. 2021; Korfmacher and Brody 2023). The Digital Exposure report back interface (DERBI) is one scalable report back tool that can generate individual GxE results with comparisons to the study group. This tool provides detailed information regarding potential sources of exposures, health effects, and strategies for exposure reduction (Korfmacher and Brody 2023; Boronow et al. 2017).

Additional innovative communication approaches, tools, and educational resources are needed to support report back of GxE findings, to evaluate effectiveness of GxE report back in different communities, and to expand GxE literacy in general. Understanding GxE concepts is critical for understanding complex disease risk and improving adoption of risk‐reduction strategies (Chen et al. 2024). Yet, the public often finds it challenging to understand how genetic and environmental influences act together to impact health outcomes (Waters, Ball, and Gehlert 2017). One recent study designed an educational intervention to communicate complex GxE concepts related to eating behavior and its influence on weight through an educational video and experiential narrative vignettes. This intervention was found to improve GxE knowledge and increase empathetic concern but did not significantly impact long‐term behavior changes (Chen et al. 2024). Another study utilized a mental model method that incorporated community engagement processes to encourage risk reduction for podoconiosis, a noninfectious lymphedema (endemic to highland Ethiopia) that is caused by a GxE interaction. This study demonstrated some of the challenges of improving GxE literacy in low‐ and middle‐income countries (Allen et al. 2019). The urgent need to communicate and apply risk modifications associated with GxE in African populations that carry an especially high burden of noncommunicable diseases associated with complex GxE findings has also been stressed (Nienaber‐Rousseau 2024). Korfmacher and Brody recently provided recommendations for guidelines, training, and resources to enhance effectiveness of reporting back individual environmental health research results and reduce barriers in report back that are applicable for GxE report back as well (Korfmacher and Brody 2023). Given that even healthcare professionals and genetic counselors can have difficulties interpreting and explaining GxE findings to patients or participants, the creation of environmental counseling programs, comparable to genetic counseling programs, has repeatedly been recognized as a need for communicating environmental and GxE report back findings. These programs could also facilitate integration of environmental health and SDOH into clinical practice.

### 4.4 Translation of GxE Results Into Action

Study participants have used report back findings for environmental exposures to inform individual, community, and policy actions to protect their health. It is anticipated that GxE study findings will be similarly informative. In one of the earliest published cases of a GxE report back, results on genetic susceptibility to arsenic toxicity in a Bangladeshi cohort resulted in an increased self‐reported behavior change to reduce arsenic exposure (Tamayo et al. 2024). All participants of this study who received report back results had expressed strong interest in receiving this information and found it useful. In addition to modifying individual behaviors, participants have shared report back results with medical providers to improve their clinical care. Lastly, participants use report back results to motivate policies to restrict environmental hazards (Brody et al. 2009; Brown et al. 2012; He, Karagas, and Murray 2018; Emmett et al. 2009; Perovich et al. 2018). For example, in one community, members switched their drinking water to avoid PFAS contaminants after testing (Emmett et al. 2009). In another study, participants became engaged in community hearings and other actions that resulted in a successful court case against an oil refinery (Brody et al. 2009; Brown et al. 2012). In some cases, report back has actually been shown to increase environmental health literacy, as well as increase community engagement and build trust between communities and researchers (Boronow et al. 2023; Brody et al. 2009; Brown et al. 2012; Hoover 2023; Perovich et al. 2018).

Several barriers can pose challenges to translating GxE research findings. A 2018 NIEHS Partnerships in Environmental Science Workshop identified “the ability to act given socioeconomic disparities” as the stand‐out challenge for empowering action—a recognition of the limits of report back as a tool for change given the lack of resources in some communities (Lebow‐Skelley et al. 2020). This sentiment is reflected in some recent report back studies. In the Environmental Influence on Childhood Health Outcomes (ECHO) Program focus groups, participants listed lack of options for alternative “safe” products, financial constraints, and limited time as barriers to reduce exposures individually or collectively, although they remained interested in learning about opportunities for action despite these barriers (Oksas et al. 2022).

Publishing scientific findings should be coupled with actionable next steps to inform change (Van Horne et al. 2023; Nigra and Navas‐Acien 2021). Researchers should help participants access resources they need to address findings involving hazardous environmental exposures or high genetic susceptibility to an environmental exposure effect. Establishing sustainable community partnerships can facilitate researchers and communities in collaboratively identifying follow‐up measures for effective intervention studies and testing strategies to promote uptake of these interventions (“NIEHS” 2023b). Consideration for how to best share research results with policy makers in a way that will enhance capacity to address the root causes of environmental exposures and inequities in communities is also needed.

## 5 Address Privacy, Discrimination, and Legal Concerns Related to GxE Findings

### 5.1 Privacy and Discrimination Concerns

GxE research raises unique privacy and discrimination concerns due to the aggregation of many data types and the limitations of current policies. Combining multiple genetic and environmental risk factors in complex studies increases the likelihood of identifiability of individuals or communities (Hammack, Brelsford, and Beskow 2019). There is concern that there is too much reliance on the Health Insurance Portability and Accountability Act (HIPAA) recommendation of “de‐identification” or anonymization of human data to protect privacy in complex human genetic and environmental population research studies. De‐identification is the process of reducing data content to decrease the likelihood of discovering a person's identity (El Emam, Rodgers, and Malin 2015). HIPAA regulations were originally designed for physician–patient relationships and are inadequate to address the complex research needs of GxE studies. Many studies have shown that the HIPAA Safe Harbor standard is likely insufficient for adequately protecting privacy in environmental health and GxE studies, because of potential ease of identifying individuals or small community areas with the breadth of environmental health data utilized, especially when combined with genetic data (Boronow et al. 2017; Boronow et al. 2020; Sweeney et al. 2017). (See Table 1 for HIPAA and HIPAA Safe Harbor references). New guidelines and best practices related to privacy concerns for GxE research are therefore needed.

Re‐identification (the process of linking “deidentified” data that lack obvious personal identifiers back to one or a few people) is also an emerging legitimate concern. Data collected in environmental health studies may overlap with public and commercial datasets or allow inference of an individual's membership in a study population, increasing risks of individual re‐identification (Boronow et al. 2020). As computational methods and artificial intelligence approaches rapidly advance, the likelihood of incidental re‐identification increases without commensurate advances in privacy protections (Sweeney et al. 2017). Re‐identification could lead to stigmas and discrimination for communities most vulnerable to environmental health harms (Boronow et al. 2020). Best practices for how researchers can simultaneously protect and share GxE research data are needed, with careful consideration of potential stigmas and discrimination concerns from known environmental exposures.

### 5.2 Unique Considerations With Geospatial Data

Integrating neighborhood environment data into GxE research will develop more accurate, context‐specific risk predictions for disease and health outcomes. However, geospatial measures (or geomarkers) can pose unique privacy challenges. Updated guidance and policies as well as new tools and approaches are needed to maximize benefits of geospatial data while protecting privacy of research participants. Tools for high resolution geomarker assessment at the population level have recently become available, but more work is needed to develop and apply these methods with appropriate privacy protections. It can be particularly challenging to work with geospatial data that additionally contains protected health information (PHI). PHI is covered by “Safe Harbor” provisions, which prohibit sharing of identifiers (e.g., names, telephone numbers, social security numbers, and so on) and quasi‐identifiers (attributes which are not identifiers themselves but can be combined with other information to identify, such as city of residence, gender, occupation, and so on) (Zandbergen 2014; Brokamp et al. 2018). Geospatial data are often collected without direct, explicit informed consent but rather as a digital byproduct of other activities. For this reason, retrospective consent is often not feasible given the nature of these data and collections. Finally, the lack of consistent messaging from institutional review boards (IRBs) regarding the use of geospatial data containing PHI further stymies review of their appropriate use (Doerr and Meeder 2022).

Approaches for sharing geospatial data containing PHI include anonymization methods (geomasking, date shifting, generalization) and the use of independent geomarker assessments, but these are generally regarded as inadequate privacy protections (Zandbergen 2014). A reproducible, standardized, and decentralized resource that researchers can use for secure, efficient, automated, and reproducible linkage of geomarkers to protected health and geolocation data is needed. The Decentralized Geomarker Assessment for Multi‐Site Studies (DeGAUSS) is one such tool recently used in the ECHO program, the Electronic Medical Records and Genomics Network (eMERGE), and other large consortia to add high resolution geospatial data for environmental exposure assessment (Brokamp et al. 2018).

### 5.3 Legal Concerns

Privacy protection of geospatial and related health data in GxE research is further stymied by the gaps in laws and regulations, such as in the Genetic Information Nondiscrimination Act (GINA). GINA addresses privacy only at the individual level; like the Common Rule that governs the protection of federally funded human subjects research, GINA does not address community privacy interests (Doerr and Meeder 2022). While GINA prohibits health insurance companies from denying insurance coverage or raising premiums due to a person's genotype and prohibits employers from firing a person or paying someone less due to their genotype, GINA narrowly applies to only a subset of employers and health insurers. State regulations governing genetic discrimination and privacy issues vary widely, creating a patchwork of legal protections and gaps across the country (Prince and Roche 2014; Anderson, Lewis, and Prince 2021). The definition of genetic data covered under GINA is quite limiting as well and does not encompass a variety of genetic data that could arise from GxE research. For example, it is unclear if epigenomics, polygenic risk scores, genetic variants associated with metabolizing a chemical (rather than associated with a disease risk), and nonhuman microbiome data (e.g., environmental microbiome) are covered under GINA.

Beyond GINA, there is no comparable legal protection afforded to environmental data, which is broad in scope and can be more sensitive to potential harms than genetic information. Compared to genetic studies, privacy risks of data sharing have not been extensively explored for environmental health or GxE studies. A more comprehensive approach to privacy protection for GxE research is needed with a growing use of genetic testing in occupational settings to identify individuals most susceptible to specific workplace exposures. For instance, numerous polymorphic metabolic genes are known to affect individual susceptibility to benzene toxicity, which has implications for occupational health risk assessment and occupational screening opportunities to protect the most susceptible individuals exposed to this chemical (Schulte, Whittaker, and Curran 2015; Carbonari et al. 2016). Many believe a worker's “Right to Know” will soon be extended to their ability to know their genetic susceptibility to workplace toxicants (Schulte, Whittaker, and Curran 2015). But a variety of concerns regarding an individual's insurability, employability, as well as privacy and confidentiality, are implicated with these additional GxE tests. There are calls for broad federal legislation and recommendations to guide such testing (Brandt‐Rauf and Brandt‐Rauf 2004; Brandt‐Rauf et al. 2011). Concerns related to increased toxic tort litigation is also a legitimate fear as knowledge regarding genetic factors involved in environmentally induced diseases grows and workers/employers clash over responsibilities and protections related to disease risks (Christiani et al. 2001 *).* GxE research may also have unique, direct financial impacts for individuals and communities. For example, there could be property value fluctuations and liability triggers for litigation based on reporting of or remediation for a regulated substance (Goho 2016).

### 5.4 Future Needs

GxE researchers (as well as researchers studying only genetic or environmental risk factors alone) should disclose risks for potential identification (or re‐identification) in informed consent documents and consider privacy risks in approaching GxE data sharing (Boronow et al. 2020). General privacy laws outside of HIPAA should be advocated since GxE research is broadening the scope of research data that could be implicated in privacy violations and discrimination. Privacy and discrimination risks to both individuals and communities should be anticipated and addressed when planning a study, but broad research consent should also be encouraged in GxE research to maximize benefit from these expensive studies. Participants could be informed that without broad consent, the ability to share, combine, and perform valuable cross study analyses with GxE data could be severely limited (“National Academies of Sciences, Engineering, and Medicine” 2020; Clayton et al. 2018). Emphasizing additional approaches to minimize risk, such as the use of a minimum necessary data standard, may also be valuable. However, the long‐term efficacy of this approach for privacy protection may be limited as GxE research, like all big data research, is increasingly shifting to hypothesis‐generating methodologies. Recognizing “de‐risk” as the goal rather than “zero risk” would be constructive; transparency with communities in terms of explaining various privacy and discrimination risks with the allowance of different levels of consent will be useful (El Emam, Rodgers, and Malin 2015; El Emam et al. 2011). For example, the Navajo Nation, aware of the risks and benefits, has welcomed biomonitoring on reservation lands to illustrate the disproportionate exposure to uranium from nuclear testing and mining waste because this knowledge is being used to seek changes in policy and push for reclamation to greatly benefit their community (Nygren 2023; Executive Order No. 04 2023).

## 6 Conclusion and Recommendations for Moving Forward

As GxE research rapidly evolves, it is important to understand and address unique ELSI that arise from this work. More resources are needed to support sustainable, authentic partnerships between researchers and communities, build community infrastructure, and provide training to conduct community‐engaged research. Additionally, a multi‐dimensional GxE research approach that integrates SDOH and incorporates new, interdisciplinary research methodologies and research teams from diverse disciplines and perspectives will be needed to conduct the most innovative and impactful GxE research. To maximize benefits and minimize potential harms from the application of GxE research findings, additional innovative tools and methods to support communication and report back are needed. A policy for report back, including guidance for quality assurance and control (particularly needed outside CLIA) and a need to further educate IRBs on the value of community engaged report‐back for environmental health and GxE studies has been recognized (“National Academies of Sciences, Engineering, and Medicine” 2018; Ohayon et al. 2017; Lebow‐Skelley et al. 2020; Brown et al. 2010). Requiring grant proposals to include a report back plan with appropriate costs and shared infrastructure should be considered. Evaluations of effectiveness of report back approaches in different communities are also needed. Following this NIH workshop, a funding opportunity was released by NIH, specifically calling for applications that explore strategies for responsibly reporting back environmental health and GxE results to research participants (“NIEHS” 2023c). NIH hopes to build on this initiative and others to continue to encourage innovation in GxE report back.

The actionability of GxE findings calls for careful consideration of how to responsibly disseminate and translate research findings and help individuals and communities use the information effectively. More research is needed to understand how people use GxE research information and how report back impacts third parties in both positive and negative ways (e.g., reporting residential lead exposures may impact landlords and neighbors in addition to residents). There is an urgent need for environmental counseling programs (analogous to genetic counseling programs), which could serve as a resource for reporting back results to participants with possible actionable preventative or intervention steps to reduce harmful exposures. Environmental counseling programs could also facilitate integration of environmental health, SDOH, and understanding of GxE into clinical settings. Furthermore, GxE studies analyzing environmental exposures in communities could support data‐informed decision‐making, resource allocation, regulations, and policies (Ackerman et al. 2017; Darling et al. 2016). Care must be taken to avoid stigmatization and discrimination of individuals and communities. Researchers could consider a solution‐oriented research approach, building upon “good science” principles with research examining modifiable risk factors, mitigation strategies, and community resilience (Senier et al. 2017; Ackerman et al. 2017).

GxE research also raises unique privacy and discrimination concerns due to the aggregation of many data types. General privacy laws outside of HIPAA and GINA should be advocated to protect both individual and community privacy and to include the broadening scope of environmental and GxE research data. GxE researchers should disclose risks for potential identification (or re‐identification) in informed consent documents and consider privacy risks in approaching GxE data sharing. Guidance on appropriate approaches to informed consent tailored to the unique features of GxE research is needed. Further, best practices for how to protect community interests (e.g., privacy, fiscal) in GxE research are necessary. Researchers should anticipate that communities may choose for their GxE data to be partially identifiable, coupled with the appropriate protections, and address this in community engagement. Careful consideration must be given for potential discrimination concerns as well as potential benefits of public awareness, particularly for communities most likely to be disproportionately affected by environmental exposures. Ultimately, a collection of new guidelines, resources, approaches, training, and capacity building are required to support innovative GxE research and multidisciplinary GxE research teams.

## Conflicts of Interest

The authors declare no conflicts of interest.

## Data Availability

Data sharing is not applicable to this article as no new data were created or analyzed in this study.

## References

[gepi22591-bib-0002] Ackerman, S. L. , K. W. Darling , S. S. J. Lee , R. A. Hiatt , and J. K. Shim . 2017. “The Ethics of Translational Science: Imagining Public Benefit in Gene‐Environment Interaction Research.” Engaging Science, Technology, and Society 3: 351–374. 10.17351/ests2017.152. 34423150 PMC8376214

[gepi22591-bib-0003] Allen, C. G. , C. M. McBride , K. Engdawork , D. Ayode , and G. Tadele . 2019. “Applying Mental Model Methods to Characterize Understanding of Gene‐Environment Influences: The Case of Podoconiosis in Ethiopia.” Critical Public Health 29, no. 1: 84–99. 10.1080/09581596.2017.1409885. 30853753 PMC6405231

[gepi22591-bib-0004] Ambrosone, C. B. , S. Kropp , J. Yang , S. Yao , P. G. Shields , and J. Chang‐Claude . 2008. “Cigarette Smoking, N‐Acetyltransferase 2 Genotypes, and Breast Cancer Risk: Pooled Analysis and Meta‐Analysis.” Cancer Epidemiology, Biomarkers & Prevention 17, no. 1: 15–26. 10.1158/1055-9965.EPI-07-0598. 18187392

[gepi22591-bib-0005] Amendola, L. M. , M. O. Dorschner , P. D. Robertson , et al. 2015. “Actionable Exomic Incidental Findings in 6503 Participants: Challenges of Variant Classification.” Genome Research 25, no. 3: 305–315. 10.1101/gr.183483.114. 25637381 PMC4352885

[gepi22591-bib-0006] Anderson, J. , A. Lewis , and A. Prince . 2021. “The Problems With Patchwork: State Approaches to Regulating Insurer Use of Genetic Information.” SSRN Electronic Journal. 10.2139/ssrn.3767117.

[gepi22591-bib-0007] Baldwin, J. A. , R. T. Trotter , M. Remiker , et al. 2021. “A Community‐Engaged Approach to Environmental Health Research: Process and Lessons Learned.” Progress in Community Health Partnerships: Research, Education, and Action 15, no. 4: 533–540. 10.1353/cpr.2021.0043. 34975035 PMC8812205

[gepi22591-bib-0008] Black, J. O. 2016. “Xeroderma Pigmentosum.” Head and Neck Pathology 10, no. 2: 139–144. 10.1007/s12105-016-0707-8. 26975629 PMC4838978

[gepi22591-bib-0009] Blacksher, E. , V. Y. Hiratsuka , J. W. Blanchard , et al. 2021. “Deliberations With American Indian and Alaska Native People About the Ethics of Genomics: An Adapted Model of Deliberation Used With Three Tribal Communities in the United States.” AJOB Empirical Bioethics 12, no. 3: 164–178. 10.1080/23294515.2021.1925775. 34125006 PMC8274345

[gepi22591-bib-0010] Bookman, E. B. , A. A. Langehorne , J. H. Eckfeldt , et al. 2006. “Reporting Genetic Results in Research Studies: Summary and Recommendations of an NHLBI Working Group.” American Journal of Medical Genetics Part A 140A: 1033–1040. 10.1002/ajmg.a.31195. PMC2556074 16575896

[gepi22591-bib-0011] Bookman, E. B. , K. McAllister , E. Gillanders , et al. 2011. “Gene‐Environment Interplay in Common Complex Diseases: Forging an Integrative Model—Recommendations From an Nih Workshop.” Genetic Epidemiology 35, no. 4: 217–225. 10.1002/gepi.20571. 21308768 PMC3228883

[gepi22591-bib-0012] Boronow, K. E. , B. Cohn , L. Havas , M. Plumb , and J. G. Brody . 2023. “The Effect of Individual or Study‐Wide Report‐Back on Knowledge, Concern, and Exposure‐Reducing Behaviors Related to Endocrine‐Disrupting Chemicals.” Environmental Health Perspectives 131, no. 9: 097005‐1. https://ehp.niehs.nih.gov/doi/10.1289/EHP12565. 37682721 10.1289/EHP12565 PMC10489892

[gepi22591-bib-0013] Boronow, K. E. , L. J. Perovich , L. Sweeney , et al. 2020. “Privacy Risks of Sharing Data From Environmental Health Studies.” Environmental Health Perspectives 128, no. 1: 017008. 10.1289/ehp4817. 31922426 PMC7015543

[gepi22591-bib-0014] Boronow, K. E. , H. P. Susmann , K. Z. Gajos , et al. 2017. “Derbi: A Digital Method to Help Researchers Offer “Right‐to‐Know” Personal Exposure Results.” Environmental Health Perspectives 125, no. 2: A27–A33. 10.1289/EHP702. 28145870 PMC5289917

[gepi22591-bib-0015] Brandt‐Rauf, P. W. , and S. I. Brandt‐Rauf . 2004. “Genetic Testing in the Workplace: Ethical, Legal, and Social Implications.” Annual Review of Public Health 25: 139–153. 10.1146/annurev.publhealth.25.101802.123012. 15015916

[gepi22591-bib-0016] Brandt‐Rauf, S. I. , E. Brandt‐Rauf , R. Gershon , and P. W. Brandt‐Rauf . 2011. “The Differing Perspectives of Workers and Occupational Medicine Physicians on the Ethical, Legal, and Social Issues of Genetic Testing in the Workplace.” NEW SOLUTIONS: A Journal of Environmental and Occupational Health Policy 21, no. 1: 89–102. 10.2190/NS.21.1.j. 21411427

[gepi22591-bib-0017] Breton, C. V. , R. Landon , and L. G. Kahn , et al. 2021. “Exploring the Evidence for Epigenetic Regulation of Environmental Influences on Child Health Across Generations.” Communications Biology 4, no. 1: 769. 10.1038/s42003-021-02316-6. 34158610 PMC8219763

[gepi22591-bib-0018] Brody, J. G. , P. M. Cirillo , K. E. Boronow , et al. 2021. “Outcomes From Returning Individual Versus Only Study‐Wide Biomonitoring Results in an Environmental Exposure Study Using the Digital Exposure Report‐Back Interface (DERBI).” Environmental Health Perspectives 129, no. 11: 117005. 10.1289/EHP9072. 34766835 PMC8589017

[gepi22591-bib-0019] Brody, J. G. , S. C. Dunagan , R. Morello‐Frosch , P. Brown , S. Patton , and R. A. Rudel . 2014. “Reporting Individual Results for Biomonitoring and Environmental Exposures: Lessons Learned From Environmental Communication Case Studies.” Environmental Health 13: 40. 10.1186/1476-069X-13-40. 24886515 PMC4098947

[gepi22591-bib-0020] Brody, J. G. , R. Morello‐Frosch , A. Zota , P. Brown , C. Pérez , and R. A. Rudel . 2009. “Linking Exposure Assessment Science With Policy Objectives for Environmental Justice and Breast Cancer Advocacy: The Northern California Household Exposure Study.” American Journal of Public Health 99, no. Suppl 3: S600–S609. 10.2105/ajph.2008.149088. 19890164 PMC2774181

[gepi22591-bib-0021] Brokamp, C. , C. Wolfe , T. Lingren , J. Harley , and P. Ryan . 2018. “Decentralized and Reproducible Geocoding and Characterization of Community and Environmental Exposures for Multisite Studies.” Journal of the American Medical Informatics Association 25, no. 3: 309–314. 10.1093/jamia/ocx128. 29126118 PMC7378876

[gepi22591-bib-0022] Brown, P. , J. G. Brody , R. Morello‐Frosch , J. Tovar , A. R. Zota , and R. A. Rudel . 2012. “Measuring the Success of Community Science: The Northern California Household Exposure Study.” Environmental Health Perspectives 120, no. 3: 326–331. 10.1289/ehp.1103734. 22147336 PMC3295345

[gepi22591-bib-0023] Brown, P. , R. Morello‐Frosch , J. G. Brody , et al. 2010. “Institutional Review Board Challenges Related to Community‐Based Participatory Research on Human Exposure to Environmental Toxins: A Case Study.” Environmental Health 9: 39. 10.1186/1476-069X-9-39. 20637068 PMC2914003

[gepi22591-bib-0024] Carbonari, D. , P. Chiarella , A. Mansi , D. Pigini , S. Iavicoli , and G. Tranfo . 2016. “Biomarkers of Susceptibility Following Benzene Exposure: Influence of Genetic Polymorphisms on Benzene Metabolism and Health Effects.” Biomarkers in Medicine 10, no. 2: 145–163. 10.2217/bmm.15.106. 26764284

[gepi22591-bib-0025] Casey, J. A. , M. Daouda , R. S. Babadi , et al. 2023. “Methods in Public Health Environmental Justice Research: a Scoping Review From 2018 to 2021.” Current Environmental Health Reports 10, no. 3: 312–336. 10.1007/s40572-023-00406-7. 37581863 PMC10504232

[gepi22591-bib-0125] Centers for Disease Control and Prevention . 2022. CLIA Law & Regulations. U.S. Department of Health & Human Services, Centers for Disease Control and Prevention. https://www.cdc.gov/clia/law-regulations.html.

[gepi22591-bib-0026] Chen, J. , A. J. Martingano , S. Ravuri , et al. 2024. “Teaching Gene‐Environment Interaction Concepts With Narrative Vignettes: Effects on Knowledge, Stigma, and Behavior Motivation.” PLOS ONE 19, no. 5: e0300452. 10.1371/journal.pone.0300452. 38722839 PMC11081345

[gepi22591-bib-0027] Christiani, D. C. , R. R. Sharp , G. W. Collman , and W. A. Suk . 2001. “Applying Genomic Technologies in Environmental Health Research: Challenges and Opportunities.” Journal of Occupational and Environmental Medicine 43, no. 6: 526–533. PMID: 11411324. 10.1097/00043764-200106000-00003. 11411324

[gepi22591-bib-0028] Claudio, L. , J. Gilmore , M. Roy , and B. Brenner . 2018. “Communicating Environmental Exposure Results and Health Information in a Community‐Based Participatory Research Study.” BMC Public Health 18, no. 1: 784. 10.1186/s12889-018-5721-1. 29940915 PMC6019712

[gepi22591-bib-0029] Claw, K. G. , M. Z. Anderson , R. L. Begay , et al. 2018. “A Framework for Enhancing Ethical Genomic Research With Indigenous Communities.” Nature Communications 9, no. 1: 2957. 10.1038/s41467-018-05188-3. PMC6063854 30054469

[gepi22591-bib-0030] Clayton, E. W. , C. M. Halverson , N. A. Sathe , and B. A. Malin . 2018. “A Systematic Literature Review of Individuals’ Perspectives on Privacy and Genetic Information in the United States.” PLoS One 13, no. 10: e0204417. 10.1371/journal.pone.0204417. 30379944 PMC6209148

[gepi22591-bib-0126] Community Partnerships to Advance Science for Society (ComPASS) . 2022. National Institutes of Health Office of Strategic Coordination ‐ The Common Fund. U.S. Department of Health & Human Services, National Institutes of Health. https://commonfund.nih.gov/compass.

[gepi22591-bib-0033] *Communicating Results: Returning Results to Participants* . 2023. (The California Department of Public Health, 2023). https://biomonitoring.ca.gov/results/communicating-results.

[gepi22591-bib-0034] Daly, B. M. , and K. A. Kaphingst . 2023. “Variability in Conceptualizations and Measurement of Genetic Literacy.” PEC Innovation 2, no. 2: 100147. 10.1016/j.pecinn.2023.100147. 37214533 PMC10194132

[gepi22591-bib-0035] Darling, K. W. , S. L. Ackerman , R. H. Hiatt , S. S. J. Lee , and J. K. Shim . 2016. “Enacting the Molecular Imperative: How Gene‐Environment Interaction Research Links Bodies and Environments in the Post‐Genomic Age.” Social Science & Medicine 155: 51–60. 10.1016/j.socscimed.2016.03.007. 26994357 PMC4815914

[gepi22591-bib-0036] Diez Roux, A. V. 2011. “Complex Systems Thinking and Current Impasses in Health Disparities Research.” American Journal of Public Health 101, no. 9: 1627–1634. 10.2105/AJPH.2011.300149. 21778505 PMC3154209

[gepi22591-bib-0037] Doerr, M. , and S. Meeder . 2022. “Big Health Data Research and Group Harm: The Scope of IRB Review.” Ethics & Human Research 44, no. 4: 34–38. 10.1002/eahr.500130. 35802789

[gepi22591-bib-0038] Drelles, K. , R. Pilarski , K. Manickam , A. B. Shoben , and A. E. Toland . 2021. “Impact of Previous Genetic Counseling and Objective Numeracy on Accurate Interpretation of a Pharmacogenetics Test Report.” Public Health Genomics 24, no. 1–2: 26–32. 10.1159/000512476. 33445171 PMC7920902

[gepi22591-bib-0039] El Emam, K. , E. Jonker , L. Arbuckle , and B. Malin . 2011. “A Systematic Review of Re‐Identification Attacks on Health Data.” PLoS One 6, no. 12: e28071. 10.1371/journal.pone.0028071. 22164229 PMC3229505

[gepi22591-bib-0040] El Emam, K. , S. Rodgers , and B. Malin . 2015. “Anonymising and Sharing Individual Patient Data.” BMJ 350: h1139. 10.1136/bmj.h1139. 25794882 PMC4707567

[gepi22591-bib-0041] Emeny, R. T. , K. Zhang , D. Goodman , et al. 2022. “Inclusion of Social and Structural Determinants of Health to Advance Understanding of Their Influence on the Biology of Chronic Disease.” Current Protocols 2, no. 10: e556. 10.1002/cpz1.556. 36200800

[gepi22591-bib-0042] Emmett, E. A. , H. Zhang , F. S. Shofer , et al. 2009. “Development and Successful Application of a “Community‐First” Communication Model for Community‐Based Environmental Health Research.” Journal of Occupational & Environmental Medicine 51, no. 2: 146–156. 10.1097/JOM.0b013e3181965d9b. 19209035 PMC3074972

[gepi22591-bib-0044] *Executive Order No. 04* ‐2023. (2023, July 18). *Addressing the Impacts of Abandoned Uranium Mines and Advocating for Immediate Action to Remediate Said Impacts*. (The Navajo Nation Office of the President, 2023). https://opvp.navajo-nsn.gov/executive-order-no-04-2023/.

[gepi22591-bib-0045] Ferryman, K. , and M. Pitcan . Data & Society and Fairness in Precision Medicine. 2018. https://datasociety.net/research/fairness-precision-medicine/.

[gepi22591-bib-0046] Finn, S. , and L. O'Fallon . 2017. “The Emergence of Environmental Health Literacy‐From Its Roots to Its Future Potential.” Environmental Health Perspectives 125, no. 4: 495–501. 10.1289/ehp.1409337. 26126293 PMC5382009

[gepi22591-bib-0047] Freeman, R. B. , and W. Huang . 2014. “Collaboration: Strength in Diversity.” Nature 513, no. 7518: 305. 10.1038/513305a. 25230634

[gepi22591-bib-0048] Garrison, N. A. , M. Hudson , L. L. Ballantyne , et al. 2019. “Genomic Research Through an Indigenous Lens: Understanding the Expectations.” Annual Review of Genomics and Human Genetics 20: 495–517. 10.1146/annurev-genom-083118-015434. 30892943

[gepi22591-bib-0049] Goho, S. A. 2016. “The Legal Implications of Report Back in Household Exposure Studies.” Environmental Health Perspectives 124, no. 11: 1662–1670. 10.1289/EHP187. 27153111 PMC5089882

[gepi22591-bib-0050] Goldenberg, A. J. , C. D. Hartmann , L. Morello , S. Brooks , K. Colón‐Zimmermann , and P. A. Marshall . 2013. “Gene‐Environment Interactions and Health Inequalities: Views of Underserved Communities.” Journal of Community Genetics 4, no. 4: 425–434. 10.1007/s12687-013-0143-3. 23494820 PMC3773320

[gepi22591-bib-0051] Gray, K. M. , and L. Martin . 2019. “Measuring Environmental Health Literacy.” In Environmental Health Literacy, edited by S. Finn , and L. O'Fallon . Cham: Springer. 10.1007/978-3-319-94108-0_2.

[gepi22591-bib-0052] Grayson, S. , M. Doerr , and J.‐H. Yu . 2020. “Developing Pathways for Community‐Led Research With Big Data: A Content Analysis of Stakeholder Interviews.” Health Research Policy and Systems 18, no. 1: 76. 10.1186/s12961-020-00589-7. 32641140 PMC7346420

[gepi22591-bib-0053] Haboush‐Deloye, A. , E. Marquez , R. Dunne , and J. R. Pharr . 2023. “The Importance of Community Voice: Using Community‐Based Participatory Research to Understand the Experiences of African American, Native American, and Latinx People during a Pandemic.” Preventing Chronic Disease 20: 220152. 10.5888/pcd20.220152. PMC10038093 36893354

[gepi22591-bib-0054] Hamilton, C. M. , L. C. Strader , J. G. Pratt , et al. 2011. “The PhenX Toolkit: Get the Most From Your Measures.” American Journal of Epidemiology 174, no. 3: 253–260. 10.1093/aje/kwr193. 21749974 PMC3141081

[gepi22591-bib-0055] Hammack, C. M. , K. M. Brelsford , and L. M. Beskow . 2019. “Thought Leader Perspectives on Participant Protections in Precision Medicine Research.” Journal of Law, Medicine & Ethics 47, no. 1: 134–148. 10.1177/1073110519840493. PMC6515916 30994064

[gepi22591-bib-0056] He, X. , M. R. Karagas , and C. Murray . 2018. “Impact of Receipt of Private Well Arsenic Test Results on Maternal Use of Contaminated Drinking Water in a U.S. Population.” Science of the Total Environment 643: 1005–1012. 10.1016/j.scitotenv.2018.06.228. 30189517 PMC8601766

[gepi22591-bib-0057] Hernick, A. D. , M. K. Brown , S. M. Pinney , F. M. Biro , K. M. Ball , and R. L. Bornschein . 2011. “Sharing Unexpected Biomarker Results With Study Participants.” Environmental Health Perspectives 119, no. 1: 1–5. 10.1289/ehp.1001988. PMC3018486 20876037

[gepi22591-bib-0058] Hiatt, R. A. , S. Z. Haslam , and J. Osuch , Breast Cancer and the Environment Research Centers . 2009. “The Breast Cancer and the Environment Research Centers: Transdisciplinary Research on the Role of the Environment in Breast Cancer Etiology.” Environmental Health Perspectives 117, no. 12: 1814–1822. 10.1289/ehp.0800120. 20049199 PMC2799453

[gepi22591-bib-0059] Hoover, A. G. 2023. “Invited Perspective: Making the Implicit Explicit‐Connecting Environmental Health Literacy and Exposure Report‐Back.” Environmental Health Perspectives 131, no. 9: 91301. 10.1289/EHP1349. 37682723 PMC10489875

[gepi22591-bib-0060] Van Horne, Y. O. , C. S. Alcala , R. E. Peltier , et al. 2023. “An Applied Environmental Justice Framework for Exposure Science.” Journal of Exposure Science & Environmental Epidemiology 33, no. 1: 1–11. 10.1038/s41370-022-00422-z. PMC8902490 35260805

[gepi22591-bib-0061] Hutter, C. M. , L. E. Mechanic , N. Chatterjee , P. Kraft , and E. M. Gillanders . 2013. “Gene‐Environment Interactions in Cancer Epidemiology: A National Cancer Institute Think Tank Report.” Genetic Epidemiology 37, no. 7: 643–657. 10.1002/gepi.21756. 24123198 PMC4143122

[gepi22591-bib-0063] Juarez, P. , P. Matthews‐Juarez , D. Hood , et al. 2014. “The Public Health Exposome: a Population‐Based, Exposure Science Approach to Health Disparities Research.” International Journal of Environmental Research and Public Health 11, no. 12: 12866–12895. 10.3390/ijerph111212866. 25514145 PMC4276651

[gepi22591-bib-0064] Kaphingst, K. A. , E. Khan , K. M. White , et al. 2021. “Effects of Health Literacy Skills, Educational Attainment, and Level of Melanoma Risk on Responses to Personalized Genomic Testing.” Patient Education and Counseling 104, no. 1: 12–19. 10.1016/j.pec.2020.07.019. 32773237 PMC7749822

[gepi22591-bib-0065] Key, K. D. , D. Furr‐Holden , E. Y. Lewis , et al. 2019. “The Continuum of Community Engagement in Research: A Roadmap for Understanding and Assessing Progress.” Progress in Community Health Partnerships: Research, Education, and Action 13, no. 4: 427–434. 10.1353/cpr.2019.0064. 31866597

[gepi22591-bib-0066] Korfmacher, K. S. , and J. G. Brody . 2023. “Moving Forward With Reporting Back Individual Environmental Health Research Results.” Environmental Health Perspectives 131, no. 12: 125002. 10.1289/EHP12463. 38095662 PMC10720702

[gepi22591-bib-0067] Kraft, P. , and H. Aschard . 2015. “Finding the Missing Gene‐Environment Interactions.” European Journal of Epidemiology 30, no. 5: 353–355. 10.1007/s10654-015-0046-1. 26026724 PMC4905576

[gepi22591-bib-0068] Krzyzanowski, M. C. , C. L. Ives , N. L. Jones , et al. 2023. “The PhenX Toolkit: Measurement Protocols for Assessment of Social Determinants of Health.” American Journal of Preventive Medicine 65, no. 3: 534–542. 10.1016/j.amepre.2023.03.003. 36935055 PMC10505248

[gepi22591-bib-0069] Ladd‐Acosta, C. , and M. D. Fallin . 2016. “The Role of Epigenetics in Genetic and Environmental Epidemiology.” Epigenomics 8, no. 2: 271–283. 10.2217/epi.15.102. 26505319

[gepi22591-bib-0070] Laurino, M. Y. , A. R. Truitt , L. Tenney , et al. 2017. “Clinical Verification of Genetic Results Returned to Research Participants: Findings From a Colon Cancer Family Registry.” Molecular Genetics & Genomic Medicine 5, no. 6: 700–708. 10.1002/mgg3.328. 29178651 PMC5702564

[gepi22591-bib-0071] Lebow‐Skelley, E. , S. Yelton , B. Janssen , E. Erdei , and M. A. Pearson . 2020. “Identifying Issues and Priorities in Reporting Back Environmental Health Data.” International Journal of Environmental Research and Public Health 17, no. 18: 6742. 10.3390/ijerph17186742. 32947900 PMC7557638

[gepi22591-bib-0072] Link, B. G. , and J. Phelan . 1995. “Social Conditions as Fundamental Causes of Disease.” Journal of Health and Social Behavior 35: 80–94. 7560851

[gepi22591-bib-0073] Lyon, G. J. , and J. P. Segal . 2013. “Practical, Ethical, and Regulatory Considerations for the Evolving Medical and Research Genomics Landscape.” Applied & Translational Genomics 2: 34–40. 10.1016/j.atg.2013.02.001. 27942444 PMC5133337

[gepi22591-bib-0074] Martin, A. R. , M. Kanai , Y. Kamatani , Y. Okada , B. M. Neale , and M. J. Daly . 2019. “Clinical Use of Current Polygenic Risk Scores May Exacerbate Health Disparities.” Nature Genetics 51, no. 4: 584–591. 10.1038/s41588-019-0379-x. 30926966 PMC6563838

[gepi22591-bib-0075] McAllister, K. , L. E. Mechanic , C. Amos , et al. 2017. “Current Challenges and New Opportunities for Gene‐Environment Interaction Studies of Complex Diseases.” American Journal of Epidemiology 186, no. 7: 753–761. 10.1093/aje/kwx227. 28978193 PMC5860428

[gepi22591-bib-0076] McNeill, L. H. , L. R. Reitzel , K. H. Escoto , et al. 2018. “Engaging Black Churches to Address Cancer Health Disparities: Project Church.” Frontiers in Public Health 6: 191. 10.3389/fpubh.2018.00191. 30073158 PMC6060541

[gepi22591-bib-0077] Mechanic, L. E. , H. S. Chen , C. I. Amos , et al. 2012. “Next Generation Analytic Tools for Large Scale Genetic Epidemiology Studies of Complex Diseases.” Genetic Epidemiology 36, no. 1: 22–35. 10.1002/gepi.20652. 22147673 PMC3368075

[gepi22591-bib-0078] Morales, E. , and D. Duffy . 2019. “Genetics and Gene‐Environment Interactions in Childhood and Adult Onset Asthma.” Frontiers in Pediatrics 7: 499. 10.3389/fped.2019.00499. 31921716 PMC6918916

[gepi22591-bib-0079] Motsinger‐Reif, A. A. , D. M. Reif , F. S. Akhtari , et al. 2024. “Gene‐Environment Interactions Within a Precision Environmental Health Framework.” Cell Genomics 4, no. 7: 100591. 10.1016/j.xgen.2024.100591. 38925123 PMC11293590

[gepi22591-bib-0080] National Academies of Sciences, Engineering, and Medicine . 2020. Applying Big Data to Address the Social Determinants of Health in Oncology: Proceedings of a Workshop. National Academies Press. 10.17226/25835. 32870621

[gepi22591-bib-0081] National Academies of Sciences, Engineering, and Medicine . 2018. Returning Individual Research Results to Participants: Guidance for a New Research Paradigm. Washington, DC: The National Academies Press. 10.17226/25094. 30001048

[gepi22591-bib-0127] National Institute of Environmental Health Sciences (NIEHS) . 2022. *About PEPH ‐ Partnerships for Environmental Public Health (PEPH)* . U.S. Department of Health & Human Services, National Institutes of Health. https://www.niehs.nih.gov/research/supported/translational/peph/about/index.cfm.

[gepi22591-bib-0128] National Institute of Environmental Health Sciences (NIEHS) . 2023a, January 11–12. *Ethical, Legal, and Social Implications of Gene‐Environment Interaction Research Virtual Workshop*. U.S. Department of Health & Human Services, National Institutes of Health. https://www.niehs.nih.gov/news/events/pastmtg/2022/elsi/index.cfm.

[gepi22591-bib-0129] National Institute of Environmental Health Sciences . 2023b. Implementation Science in Environmental Health ‐ Promoting Evidence‐based Prevention and Intervention to Advance Environmental Health Equity. U.S. Department of Health & Human Services, National Institutes of Health. https://www.niehs.nih.gov/research/supported/translational/implementation/index.cfm.

[gepi22591-bib-0130] National Institute of Environmental Health Sciences . 2023c. Strategies for Responsibly Reporting Back Environmental Health and Non‐Genomic Research Results (R01 Clinical Trial Optional). U.S. Department of Health & Human Services, National Institutes of Health. https://grants.nih.gov/grants/guide/rfa-files/RFA-ES-23-006.html.

[gepi22591-bib-0131] National Institutes of Health All of Us Research Program . 2023. Social Determinants of Health Survey. U.S. Department of Health & Human Services, National Institutes of Health. https://www.researchallofus.org/data-tools/survey-explorer/social-determinants-survey/.

[gepi22591-bib-0132] National Institute on Minority Health and Health Disparities . 2023. National Minority Health and Health Disparities Research Framework. U.S. Department of Health & Human Services, National Institutes of Health. https://www.nimhd.nih.gov/about/overview/research-framework/.

[gepi22591-bib-0133] National Institute on Minority Health and Health Disparities . 2020. Notice Announcing Availability of Data Harmonization Tools for Social Determinants of Health (SDOH) via the PhenX Toolkit. U.S. Department of Health & Human Services, National Institutes of Health. https://grants.nih.gov/grants/guide/notice-files/NOT-MD-21-003.html.

[gepi22591-bib-0083] Nienaber‐Rousseau, C. 2024. “Understanding and Applying Gene‐Environment Interactions: A Guide for Nutrition Professionals With an Emphasis on Integration in African Research Settings.” Nutrition Reviews: nuae015. 10.1093/nutrit/nuae015. 38442341 PMC11723160

[gepi22591-bib-0084] Nigra, A. E. , and A. Navas‐Acien . 2021. “Racial Inequalities in Drinking Water Lead Exposure: A Wake‐Up Call to Protect Patients With End Stage Kidney Disease.” Journal of the American Society of Nephrology 32, no. 10: 2419–2421. 10.1681/asn.2021060793. 34544822 PMC8722796

[gepi22591-bib-0086] Nygren, B. (2023). The Navajo Suffered From Nuclear Testing. Oppenheimer Doesn't Tell Our Story. TIME. https://time.com/6296470/oppenheimer-navajo-uranium-mining-essay/.

[gepi22591-bib-0087] Ohayon, J. L. , E. Cousins , P. Brown , R. Morello‐Frosch , and J. G. Brody . 2017. “Researcher and Institutional Review Board Perspectives on the Benefits and Challenges of Reporting Back Biomonitoring and Environmental Exposure Results.” Environmental Research 153: 140–149. 10.1016/j.envres.2016.12.003. 27960129 PMC5412511

[gepi22591-bib-0088] Oksas, C. , J. G. Brody , P. Brown , Program Collaborators for Environmental Influences on Child Health Outcomes , et al. 2022. “Perspectives of Peripartum People on Opportunities for Personal and Collective Action to Reduce Exposure to Everyday Chemicals: Focus Groups to Inform Exposure Report‐Back.” Environmental Research 212, no. Pt A: 113173. 10.1016/j.envres.2022.113173. 35351450 PMC9244766

[gepi22591-bib-0089] Pang, S. , L. Yengo , C. P. Nelson , et al. 2023. “Genetic and Modifiable Risk Factors Combine Multiplicatively in Common Disease.” Clinical Research in Cardiology 112, no. 2: 247–257. 10.1007/s00392-022-02081-4. 35987817 PMC9898372

[gepi22591-bib-0090] Perovich, L. J. , J. L. Ohayon , E. M. Cousins , et al. 2018. “Reporting to Parents on Children's Exposures to Asthma Triggers in Low‐Income and Public Housing, an Interview‐Based Case Study of Ethics, Environmental Literacy, Individual Action, and Public Health Benefits.” Environmental Health 17, no. 1: 48. 10.1186/s12940-018-0395-9. 29784007 PMC5963109

[gepi22591-bib-0091] PhenX Toolkit: Collections . www.phenxtoolkit.org. (2024). https://www.phenxtoolkit.org/collections/view/6.

[gepi22591-bib-0092] Polka, E. , E. Childs , A. Friedman , et al. 2021. “MCR: Open‐Source Software to Automate Compilation of Health Study Report‐Back.” International Journal of Environmental Research and Public Health 18, no. 11: 6104. 10.3390/ijerph18116104. 34198866 PMC8201126

[gepi22591-bib-0093] Prince, A. E. R. , and M. I. Roche . 2014. “Genetic Information, Non‐Discrimination, and Privacy Protections in Genetic Counseling Practice.” Journal of Genetic Counseling 23, no. 6: 891–902. 10.1007/s10897-014-9743-2. 25063358 PMC4233176

[gepi22591-bib-0094] Ramirez‐Andreotta, M. , J. Brody , N. Lothrop , M. Loh , P. Beamer , and P. Brown . 2016. “Improving Environmental Health Literacy and Justice Through Environmental Exposure Results Communication.” International Journal of Environmental Research and Public Health 13, no. 7: 690. 10.3390/ijerph13070690. 27399755 PMC4962231

[gepi22591-bib-0095] Rasnick, E. , P. Ryan , J. Blossom , et al. 2023. “High Resolution and Spatiotemporal Place‐Based Computable Exposures at Scale.” AMIA Joint Summits on Translational Science proceedings. AMIA Joint Summits on Translational Science 2023: 62–70. 37350915 PMC10283107

[gepi22591-bib-0096] Ray, K. , and J. F. Cooper . 2024. “The Bioethics of Environmental Injustice: Ethical, Legal, and Clinical Implications of Unhealthy Environments.” The American Journal of Bioethics 24, no. 30: 9–17. 10.1080/15265161.2023.2201192. 37104666

[gepi22591-bib-0097] Ribatti, D. 2021. “Epigenetics and Its Ethical Implications.” Critical Reviews in Eukaryotic Gene Expression 31, no. 1: 23–27. 10.1615/CritRevEukaryotGeneExpr.2020036701. 33639052

[gepi22591-bib-0098] Ritz, B. R. , N. Chatterjee , M. Garcia‐Closas , et al. 2017. “Lessons Learned From Past Gene‐Environment Interaction Successes.” American Journal of Epidemiology 186, no. 7: 778–786. 10.1093/aje/kwx230. 28978190 PMC5860326

[gepi22591-bib-0099] Sanderson, S. , J. Emery , and J. Higgins . 2005. “CYP2C9 Gene Variants, Drug Dose, and Bleeding Risk in Warfarin‐Treated Patients: A HuGEnet Systematic Review and Meta‐Analysis.” Genetics in Medicine 7, no. 2: 97–104. 10.1097/01.gim.0000153664.65759.cf. 15714076

[gepi22591-bib-0100] Santaló, J. , and M. Berdasco . 2022. “Ethical Implications of Epigenetics in the Era of Personalized Medicine.” Clinical Epigenetics 14: 44. 10.1186/s13148-022-01263-1. 35337378 PMC8953972

[gepi22591-bib-0101] Saulnier, K. , A. Berner , S. Liosi , et al. 2022. “Studying Vulnerable Populations Through an Epigenetics Lens: Proceed With Caution.” Canadian Journal of Bioethics/Revue canadienne de bioéthique 5, no. 1: 68–78. 10.7202/1087205ar.

[gepi22591-bib-0102] Schulte, P. A. , C. Whittaker , and C. P. Curran . 2015. “Considerations for Using Genetic and Epigenetic Information in Occupational Health Risk Assessment and Standard Setting.” Journal of Occupational and Environmental Hygiene 12, no. sup1: S69–S81. 10.1080/15459624.2015.1060323. 26583908 PMC4685594

[gepi22591-bib-0103] Science Benefits From Diversity . (2018). Nature, 558 (7708), 5. 10.1038/d41586-018-05326-3. 31076730

[gepi22591-bib-0104] Senier, L. , P. Brown , S. Shostak , and B. Hanna . 2017. “The Socio‐Exposome: Advancing Exposure Science and Environmental Justice in a Post‐Genomic Era.” Environmental Sociology 3, no. 2: 107–121. 10.1080/23251042.2016.1220848. 28944245 PMC5604315

[gepi22591-bib-0107] Suckiel, S. A. , J. M. O'Daniel , K. E. Donohue , et al. 2021. “Genomic Sequencing Results Disclosure in Diverse and Medically Underserved Populations: Themes, Challenges, and Strategies From the CSER Consortium.” Journal of Personalized Medicine 11, no. 3: 202. 10.3390/jpm11030202. 33805616 PMC7998798

[gepi22591-bib-0108] Sweeney, L. , J. S. Yoo , L. Perovich , K. E. Boronow , P. Brown , and J. G. Brody . 2017. “Re‐Identification Risks in HIPA Safe Harbor Data: A Study of Data From One Environmental Health Study.” Technology Science 2017: 2017082801. 30687852 PMC6344041

[gepi22591-bib-0109] Tamayo, L. I. , S. E. Haque , T. Islam , et al. 2024. “Returning Personal Genetic Information on Susceptibility to Arsenic Toxicity to Research Participants in Bangladesh.” Environmental Research 240, no. Pt 2: 117482. 10.1016/j.envres.2023.117482. 37879393 PMC10842833

[gepi22591-bib-0110] Thomas, D. 2010. “Gene‐Environment‐Wide Association Studies: Emerging Approaches.” Nature Reviews Genetics 11, no. 4: 259–272. 10.1038/nrg2764. PMC2891422 20212493

[gepi22591-bib-0111] Tomsho, K. S. , E. Polka , S. Chacker , et al. 2022a. “Characterizing the Environmental Health Literacy and Sensemaking of Indoor Air Quality of Research Participants.” International Journal of Environmental Research and Public Health 19, no. 4: 2227. 10.3390/ijerph19042227. 35206415 PMC8871841

[gepi22591-bib-0112] Tomsho, K. S. , E. Polka , S. Chacker , et al. 2022b. “A Process for Creating Data Report‐Back Tools to Improve Equity in Environmental Health.” Environmental Health 21, no. 1: 67. 10.1186/s12940-022-00880-w. 35821055 PMC9277935

[gepi22591-bib-0113] Tomsho, K. S. , C. Schollaert , T. Aguilar , et al. 2019. “A Mixed Methods Evaluation of Sharing Air Pollution Results With Study Participants via Report‐Back Communication.” International Journal of Environmental Research and Public Health 16, no. 21: 4183. 10.3390/ijerph16214183. 31671859 PMC6862165

[gepi22591-bib-0114] Varma, T. , C. P. Jones , C. Oladele , and J. Miller . 2023. “Diversity in Clinical Research: Public Health and Social Justice Imperatives.” Journal of Medical Ethics 49, no. 3: 200–203. 10.1136/medethics-2021-108068. 35428737

[gepi22591-bib-0115] Virolainen, S. J. , A. VonHandorf , K. Viel , M. T. Weirauch , and L. C. Kottyan . 2023. “Gene‐Environment Interactions and Their Impact on Human Health.” Genes and Immunity 24, no. 1: 1–11. 10.1038/s41435-022-00192-6. 36585519 PMC9801363

[gepi22591-bib-0116] Wallerstein, N. B. , and B. Duran . 2006. “Using Community‐Based Participatory Research to Address Health Disparities.” Health Promotion Practice 7, no. 3: 312–323. 10.1177/1524839906289376. 16760238

[gepi22591-bib-0117] Wang, C. , K. A. Bertrand , M. Trevino‐Talbot , et al. 2023. “Ethical, Legal, and Social Implications (ELSI) and Challenges in the Design of a Randomized Controlled Trial to Test the Online Return of Cancer Genetic Research Results to U.S. Black Women.” Contemporary Clinical Trials 132: 107309. 10.1016/j.cct.2023.107309. 37516165 PMC10544717

[gepi22591-bib-0118] Waters, E. A. , L. Ball , and S. Gehlert . 2017. ““I Don't Believe It.” Acceptance and Skepticism of Genetic Health Information Among African American and White Smokers.” Social Science & Medicine (1982) 184: 153–160. 10.1016/j.socscimed.2017.04.053. 28527373 PMC5535773

[gepi22591-bib-0119] Wynn, J. , K. Lewis , L. M. Amendola , et al. 2018. “Clinical Providers’ Experiences With Returning Results From Genomic Sequencing: An Interview Study.” BMC Medical Genomics 11, no. 1: 45. 10.1186/s12920-018-0360-z. 29739461 PMC5941324

[gepi22591-bib-0120] Zandbergen, P. A. 2014. “Ensuring Confidentiality of Geocoded Health Data: Assessing Geographic Masking Strategies for Individual‐Level Data.” Advances in Medicine 2014: 1–14. 10.1155/2014/567049. PMC4590956 26556417

[gepi22591-bib-0121] Zota, A. R. , and B. N. VanNoy . 2021. “Integrating Intersectionality into the Exposome Paradigm: A Novel Approach to Racial Inequities in Uterine Fibroids.” American Journal of Public Health 111, no. 1: 104–109. 10.2105/ajph.2020.305979. 33211578 PMC7750596

